# Acute Effects of Resistance Exercise and the Use of GH or IGF-1 Hormones on Oxidative Stress and Antioxidant Markers in Bodybuilders

**DOI:** 10.3390/antiox8120587

**Published:** 2019-11-26

**Authors:** Heidar Mohammadjafari, Hamid Arazi, Nematollah Nemati, Tahereh Bagherpoor, Katsuhiko Suzuki

**Affiliations:** 1 Department of Sport Physiology, Islamic Azad University of Damghan, Semnan 3671639998, Iran; toofanmj@yahoo.com (H.M.); nnemati258@gmail.com (N.N.); bagherpoor_ta@yahoo.com (T.B.); 2 Department of Exercise Physiology, Faculty of Sport Sciences, University of Guilan, Rasht 4199843653, Iran; 3 Faculty of Sport Sciences, Waseda University, Tokorozawa 359-1192, Japan; katsu.suzu@waseda.jp

**Keywords:** reactive oxygen species (ROS), strength exercise, insulin-like growth factor-1 (IGF-1), growth hormone (GH), stress

## Abstract

The aim of this study was to examine the influence of peptide hormone use on oxidative stress and antioxidant responses to a single session of resistance exercise in male bodybuilders. Forty-five male bodybuilders were divided into three groups: bodybuilders using growth hormone for at least 1 year (i.e., 3 to 4 times in the year) (GH-user, *n* = 15), bodybuilders using insulin-like growth factor-1 for at least 1 year (i.e., 3 to 4 times in the year) (IGF-1-user, *n* = 15), and peptide hormone-free bodybuilders (Non-user, *n* = 15). The heavy resistance exercise protocol consisted of five sets with 80% of one repetition maximum for six exercises. Blood samples were obtained pre and post heavy resistance exercise (HRE) in order to evaluate changes in oxidative stress (8-hydroxy-2-deoxyguanosine (8-OHdG), malondialdehyde (MDA), and nitric oxide (NO)) and antioxidant biomarkers (glutathione peroxidase (GPx), catalase (CAT) and glutamate (GLU)) level. All the experimental groups showed increases in MDA (*p* = 0.038), NO (*p* = 0.028), GPx (*p* = 0.012), and GLU (*p* = 0.003) concentrations after resistance exercise. For 8-OHdG, the Non-user and IGF-1-user groups indicated increases at post-exercise (*p* = 0.001), without any significant changes in the GH-user group (*p* = 0.87). In addition, the changes in serum GPx and GLU levels were greater for the GH-user group than the Non-user and IGF-1-user groups (*p* = 0.001). In conclusion, HRE induced significant increases in 8-OHdG (except to GH-user group), MDA, NO, GPx, and GLU levels with greater changes in GPx and GLU for the GH-user group.

## 1. Introduction

Use of peptide hormones has increased among athletes especially in some bodybuilders to improve lean body mass or fat free mass, muscle size, protein synthesis and decrease body fat [1]. Hoffman et al. [2] proposed that growth hormone (GH), also known as somatotropin, is an anabolic hormone which plays an important role to build and repair tissue throughout the body. In addition, insulin-like growth factor 1 (IGF-1) is another peptide hormone which can increase muscle mass and also helps to facilitate the body’s response to exercise [3].

Although amounts of some hormones may be useful in some pathological conditions, injections of peptide hormones (i.e., GH and IGF-1) are widely abused by some athletes and bodybuilders to enhance performance and for this goal World Anti-Doping Agency (WADA) prohibited the use of peptide hormones such as GH more than clinical and therapeutic conditions [1,4]. However, there is not enough evidence to show the potentially adverse effects of peptide hormones abuse on the hepatic, endocrine, cardiovascular and immune systems. In clinical dosage, Liu et al. [4] in a systematic review reported that use of GH could improve body composition and increased lean mass following resistance training intervention. Meinhardt et al. [5] examined the effect of a 2 mg dose of GH on body composition and performance adaptations and found that resistance training plus GH injection increased athletic exercise performance and participants that received GH retained more body fluid and had more frequent joint pain than the control group.

Collectively, GH and IGF-1 have anti-catabolic and anabolic effects and for this goal some athletes and bodybuilders have used peptide hormones to increase beneficial effects of training [1]; however, adverse effects of peptide hormones on immune function following resistance exercise are unknown. In fact, bodybuilders performed heavy resistance exercise and it could induce oxidative stress with increasing free radicals and reactive oxygen species (ROS). On the other hand, intense resistance exercise has been shown to increase the circulating concentrations of neutrophils, elastase, and myeloperoxidase, resulting in free radical production, oxidative stress, and inflammation [6,7]. 

To the authors’ knowledge, very few studies have examined the effects of peptide hormone usage on oxidative stress and antioxidant biomarkers after resistance exercise in male bodybuilders. Based upon the beneficial effects of GH and IGF-1 use for elevating muscle size and hypertrophy [4,5,8], limited research exists examining another physiological effects of peptide hormone use in bodybuilders. It seems that use of peptide hormones can impact cell metabolism through resistance exercise; however, in literature, little is known concerning the influence of peptide hormone (i.e., GH and IGF-1) use on oxidative stress and antioxidant responses to resistance exercise. Therefore, the aim of this study was to examine the effects of peptide hormones (i.e., GH and IGF-1) use on oxidative stress (8-hydroxy-2-deoxyguanosine (8-OHdG), malondialdehyde (MDA), and nitric oxide (NO)) and antioxidant biomarkers (glutathione peroxidase (GPx), catalase (CAT) and glutamate (GLU)) in responses to heavy resistance exercise in bodybuilders. 

## 2. Materials and Methods

### 2.1. Experimental Design

A cross-sectional design was used in the study. The participants were recruited to the laboratory four times and standardized tests during a period of four days were scheduled by 24 -h intervals and were completed at the same time of the day (i.e., 5 p.m) and by the same investigator through intervention period. On day one, the participants were familiarized with testing and resistance exercise procedures and their height and body mass were measured. On day two, the participants performed one repetition of maximum (1RM) test for the bench press, rowing, and knee extension exercises. On day three, the 1RM test for the back squat, arm curl, and knee flexion were measured. On day four (72 h after day three), the participants performed a single session of heavy resistance exercise (HRE) in the afternoon (5 pm). Pre and post HRE blood samples were obtained to analyze changes in oxidative stress and antioxidant biomarkers (i.e., 8-OHdG, MDA, NO, GPx, CAT, GLU). The test-retest reliability of this study procedure was 0.96.

### 2.2. Participants

**Figure 1:**
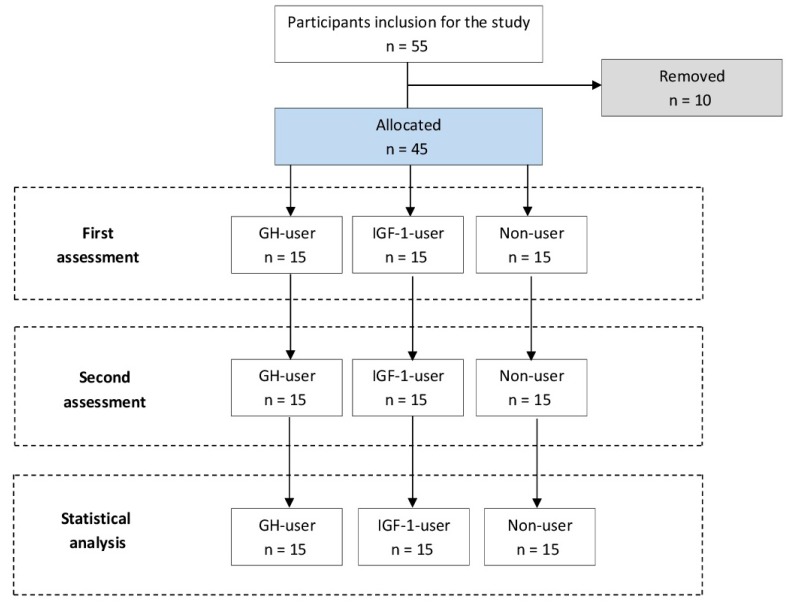
Study flow.

Initially, 55 male bodybuilders from local gyms volunteered to participate in the study. To be included in the study, participants were required to complete all measurements and have inclusion criteria to the study. As a result of these requirements, 10 subjects were removed from the study. Therefore, 45 male bodybuilders were included in the study and were allocated into 3 groups: bodybuilders using growth hormone for at least 1 year (GH-user, *n* = 15), bodybuilders using insulin-like growth factor-1 for at least 1 year (IGF-1-user, *n* = 15), and peptide hormone-free bodybuilders (Non-user, *n* = 15) (Table 1). The male bodybuilders fulfilled the following inclusion criteria: (a) between 25 and 40 years old, (b) at least 3 years’ experience in resistance training program for three to five training sessions per week, (c) no background in lower and upper body injuries, or musculoskeletal disorders and surgery, (d) no background in cardiovascular, metabolic and respiratory diseases which was checked by a physician (Figure 1). The peptide hormone-using groups were self-administrating GH or IGF-1 in a cyclic fashion (three to ten mcg daily, three to four times during the one year with two months of washout between periods). Institutional review board approval (IAU-9031397) for our study was obtained by Islamic Azad university, and the participants were informed about procedures of the study and advantages or possible hazards which are associated following the participation to the study, and each subject signed an informed consent form before participation in the study. 

### 2.3. Measurements 

Height was measured using a wall mounted stadiometer (Seca 222, Terre Haute, IN, United States) recorded to the nearest 1 cm; body mass was measured to the nearest 0.5 kg using a digital scale (Tanita, BC-418MA, Tokyo, Japan); and body mass index was calculated (kg m^−2^).

### 2.4. Muscle Strength Assessment 

The 1RM for the exercises (i.e., bench press, lat rowing, arm curl, back squat, knee extension and knee flexion) was measured to design of heavy resistance exercise (HRE). The 1RM testing procedures were carried out based on the Haff and Triplett guidelines [9]. For more safety and to ensure participants strives to volitional exhaustion, spotters attended throughout the testing and exercise sessions.

### 2.5. Resistance Exercise Program

The resistance exercise (RE) program was monitored by an experienced strength and conditioning coach to control training variables (i.e., rest, training intensity or tempo of movement). Before the HRE, each subject started to warming-up activities consisted of ten minutes cycle ergometry, five minutes stretching, and five minutes movements. Then, each subject performed specific to weight training warm-up including one set of five repetitions with intensity 50–60% of 1RM before each exercise. The participants completed HRE including 5 sets with 80% of 1RM to failure for the back squat, knee extension, knee flexion, lat rowing, bench press, and arm curl, respectively. The rest intervals between exercises and sets were 30 and 60 sec, respectively.

### 2.6. Blood Measurement and Analyzes 

Blood samples were taken at pre- (i.e., approximately 5-min to exercise beginning) and post HRE (i.e., within 5 min after terminating the exercise session) to monitor the effects of RE on oxidative and antioxidant markers. Amount of 5-mL blood sample was extracted from an antecubital vein by venipuncture. The obtained samples were kept at natural condition and room temperature for 30 min to ensure clotting and then centrifuged at velocity of 1500× *g* for 10 min. The serum layer was eliminated and placed in freezer environment at –20 °C and finally used for further analyses.

Commercially available, enzyme-linked immunosorbent assay (ELISA) kits (Eastbiopharm Co., Ltd., Hangzhou., China) were used for analysis of serum 8-hydroxy-2-deoxyguanosine (8-OHdG), nitric oxide (NO), and malondialdehyde (MDA). The assay was carried out in duplicate and intra-assay coefficients of variances were < 5% for all blood measurements. Serum antioxidant biomarker levels (glutathione peroxidase (GPx), catalase (CAT) and glutamate (GLU)) were analyzed using commercially available, ELISA kits (Eastbiopharm Co., Ltd., Hangzhou., China). The assay was carried out in duplicate and intra-assay coefficients of variances were < 7% for all blood measurements.

### 2.7. Diet Control

In order to control the influence of dietary daily intake on oxidative stress and antioxidant marker variables, 3 consecutive days of diet recall were completed before beginning the initiation of the study and presented in Table 2.

### 2.8. Statistical Analysis

All data are presented as mean ± standard deviation (SD). The 3 (groups) × 2 (times) ANOVA was used to determine the effect of HRE on oxidative stress and antioxidant changes. When a significant *F* value was obtained, Bonferroni’s post-hoc test was carried out to discriminate the possible differences between the means. For each measure, a percent change score was calculated ((post–pre)/pre × 100). The significance level was set at *p* ≤ 0.05.

## 3. Results

There were no significant differences among groups in dependent variables at pre-test (*p* > 0.05). The HRE induced a significant change in 8-OHdG biomarker levels for the Non-user and IGF-1-user groups (*F* = 3.08, *p* = 0.001), without any significant change for the GH-user group (*F* = 0.13, *p* = 0.87). Likewise, the Non-user group also indicated significant differences as compared to the GH-user group in 8-OHdG (*F* = 2.96, *p* = 0.033) at post-HRE (Table 3).

The HRE induced a significant elevation in MDA biomarker levels for all the groups (*p* = 0.038), and there were no statistically significant differences among the groups after HRE (*F* = 4.23, *p* = 0.37); however, the percentage of change in MDA were greater for the Non-user (16.7%) and IGF-1-user (18.2%) groups than GH-user (5.9%) group and these differences were not significant (Table 3).

All the groups indicated significant increases in NO levels from pre to post-HRE (*F* = 3.25, *p* = 0.028). The HRE induced a significant elevation in GPx biomarker levels for all the groups (*F* = 4.8, *p* = 0.012). The GH group also indicated significant differences as compared to the Non-user and IGF-1 groups in GPx (*F* = 5.5, *p* = 0.007) at post-HRE.

As for the CAT, no significant group difference by time interaction effect was observed (*F* = 0.481, *p* = 0.621) and no statistically significant changes were seen from pre to post-HRE for all the groups (*p* < 0.05) (Table 3).

The HRE induced a significant elevation in GLU biomarker levels for all the groups (*F* = 8.23, *p* = 0.003). The GH group also indicated significant differences as compared to the Non-user and IGF-1 groups in GLU (*F* = 9.42, *p* = 0.001) at post-HRE (Table 3).

## 4. Discussion

The aim of this study was to examine the influence of peptide hormone use on oxidative stress and antioxidant marker responses to HRE in male bodybuilders. We found that HRE induced increases in 8-OHdG, MDA, NO, GPx and GLU levels at post-exercise for all the groups (except 8-OHdG for the GH-user group). In addition, the changes in 8-OHdG were greater in the Non-user group, and GPx and GLU levels were greater for the GH-user group than other groups.

In relation to changes in oxidative stress biomarkers, 8-OHdG as a biomarker of oxidative DNA damage, MDA as a biomarker of lipid peroxidation, and NO were evaluated [10,11,12]. All the experimental groups increased the 8-OHdG (except the GH-user group), MDA and NO levels at post-HRE, likewise the Non-user group, indicated more changes than the IGF-1-user group in the 8-OHdG level following exercise intervention. In line with increases in oxidative stress levels at post-exercise, Radak et al. [13] reported that the level of 8-OHdG increased significantly after 200 eccentric contractions with knee extensors. Ramel et al. [14] reported the increases in MDA levels post-RE and increases in MDA level after different intensities and types of exercise have been reported by other studies [6,7,10,11]. The possible explanation for increases in 8-OHdG and MDA levels could be due to xanthine-xanthine oxidase pathway, changes in calcium homeostasis, increases in catecholamine autoxidation, and ischemia reperfusion by RE for 8-OHdG and cellular membranes damage following HRE to increase MDA for the treatment groups [6,7]. In fact, cell membranes have polyunsaturated fatty acid properties and HRE increased concentration of polyunsaturated fatty acids in the blood resulting in increases of lipid peroxidation (i.e., MDA) after exercise in the blood [11]. For NO, enhancements after HRE for the treatment groups are in line with previous studies which reported increases in NO after physical exercise [12]. The HRE which increased the blood flow may produce shear stress resulting in endogenous NO formation and also enhancements of NO in the blood.

To the authors’ knowledge, it is the first study that examined the influence of peptide hormone use on oxidative stress responses to HRE in male bodybuilders and for this reason the discussion could be limited. In this study, the changes in 8-OHdG were greater for the Non-user group than IGF-1-user group without any changes in the GH-user group. It seems that use of GH induced greater antioxidant capacities (Table 3, greater GPx and GLU levels at post-HRE) for the GH-user group which could be a mechanism to prevent change in 8-OHdG level following exercise intervention. In addition, the changes in NO levels were greater for the peptide hormone user groups (but not statistically significant). It seems that prolonged use of peptide hormone induced cellular adaption to control free radicals throughout resistance exercise in bodybuilders who used GH or IGF-1 hormones. In addition, injection of these hormones helps the body to angiogenesis by enhancing NO levels produced by GH-IGF-1 pathway [15]. However, the data about this subject are little and more studies are necessary to clarify the role of peptide hormone use on oxidative stress markers. 

In relation to the changes in antioxidant biomarkers, CAT and GPx as the primary defense against ROS, and GLU were evaluated [16]. All the experimental groups increased the GPx and GLU levels at post-HRE, with greater elevations for the GH-user group than other groups without any changes in CAT for the groups at post-HRE. In line with our findings, previous studies reported that a session of physical exercise could increase the levels of GPx and GLU at post-exercise [14,17]; however, other studies reported conflicting results [18,19] and the possible explanation for this discrepancy could be due to differences in exercise type and intensity, subject characteristics, the ability of subjects to perform each exercise, and environmental conditions that affect the occurrence of antioxidant levels in the body. In fact, when HRE induced ROS and free radical production in the body, the defense system is induced by enhancing GPx as the primary defense against ROS generated during exercise and GLU levels help to control cell oxidant/antioxidant status [7,14]. On the other hand, greater oxidative damage and enhancement of catecholamines are in accordance with higher antioxidant activity produced as an auto-defense mechanism following RE [16,17]. 

The greater GPx and GLU levels at post-HRE for the GH-user group indicate more antioxidant ability of GH use in bodybuilders. On the other hand, long time use of peptide hormones such as GH may produce more oxidative status in the body, resulting in greater antioxidant capacity for controlling cell metabolism and also more GPx and GLU levels following the HRE [16]. In addition, peptide hormone treatment for long years may increase overproduction of ROS and exceeding antioxidant defenses systems against oxidative damage following resistance exercise; however, more studies are needed to clarify the role of GH or IGF-1 injections on symptoms of oxidative and antioxidant activity produced by RE. 

The limitations of this study include the low number of male bodybuilders which diminish generalization of the results. Additionally, the results of the current investigation are based on the male population, and further research is needed to determine if similar effects are obtained in the female subjects. In addition, we had some limitations to increase other blood oxidative and antioxidant variables such as total antioxidant capacity or protein carbonyl and/or other peptide hormone use by athletes such as growth hormone releasing peptide (GHRP-6), GHRP-2, Hexarelin, and etc. Therefore, more studies are necessary to clarify the other aspects of peptide hormone use on oxidative stress variables.

## 5. Conclusions

In conclusion, HRE induced significant increases in 8-OHdG (except to GH-user group), MDA, and NO. In addition, the responses of GPx and GLU levels at post-exercise were higher for the GH-user group than the Non-user or IGF-1-user groups. The present study has not reported a range of negative redox status consequence of peptide hormone use in conjunction with resistance training. On the other hand, GH use for at least one year in bodybuilders induced greater antioxidant capacity after HRE. It can be considered that peptide hormone use by bodybuilders did not induce further changes in oxidative stress markers but induced greater changes related to antioxidant markers following resistance exercise.

## 6. Clinical Implication

Use of peptide hormones such as GH and/or IGF-1 has a potential effect on the oxidative stress and antioxidant capacity of the male bodybuilders. Collectively, given impacts on GLU and GPx the use of GH peptide hormone could induce the antioxidant defense system following single bout of HRE.

## Figures and Tables

**Table 1:** Subjects’ characteristics. Data are presented as mean ± SD.

Variable	Non-User	GH-User	IGF-1-User
Age (y)	27.6 ± 4.8	28.9 ± 6.3	28.6 ± 6.4
Height (cm)	180 ± 6	181 ± 8	181 ± 7
Body mas (kg)	90.1 ± 10.7	90.7 ± 10.5	94.9 ± 9.2
BMI (kg m^−2^)	28.6 ± 4.2	27.9 ± 4.4	29.3 ± 3.7
1RM bench press (kg)	105.4 ± 11.2	100.7 ± 10.6	110.2 ± 9.7
1RM rowing (kg)	64.8 ± 9.3	60.1 ± 7.5	69.1 ± 10.5
1RM arm curl (kg)	58.6 ± 7.1	55.3 ± 4.6	60.4 ± 7.6
1RM back squat (kg)	128.6 ± 9.9	120.3 ± 8.1	130.7 ± 12.9
1RM knee extension (kg)	98.6 ± 6.2	93.1 ± 9.6	101.3 ± 8.3
1RM knee flexion (kg)	57.8 ± 10.7	53.1 ± 12.3	61.4 ± 12.6

Notes: BMI: body mass index; 1RM: one repetition maximum; GH: growth hormone; IGF-1: insulin-like growth factor-1.

**Table 2:** Dietary daily intake assessed for the groups. Data are presented as mean ± SD.

Variable	Non-User	GH-User	IGF-1-User
Energy intake (kcal)	3132 ± 210	2992 ± 176	3011 ± 318
Carbohydrate (g)	293 ± 43	289 ± 39	299 ± 51
Fat (g)	95 ± 20	97 ± 19	96 ± 21
Protein (g)	130 ± 21	132 ± 24	135 ± 16
Vitamin E (mg)	9.9 ± 1.0	9.7 ± 1.9	10.7 ± 1.6
Vitamin C (mg)	71 ± 20	70 ± 17	74 ± 22

**Table 3:** Changes in the variables for the treatment groups following heavy resistance exercise (HRE).

Variable	Groups	Pre-Test	Post-Test	% of Change
**8-OHdG**	Non-user	58.0 ± 11.9	71.4 ± 15.5 *†	23.1
**(ng/mL)**	GH-user	59.1 ± 11.1	59.2 ± 10.1	0.1
	IGF-1-user	56.7 ± 10.1	69.1 ± 12.2 *	21.8
**MDA**	Non-user	50.1 ± 9.5	58.5 ± 10.1 *	16.7
**(ng/mL)**	GH-user	54.2 ± 7.2	57.4 ± 10.2 *	5.9
	IGF-1-user	52.6 ± 8.8	62.2 ± 9.2 *	18.2
**NO**	Non-user	4.1 ± 1.3	4.5 ± 1.5 *	9.7
**(μM)**	GH-user	4.6 ± 1.1	5.6 ± 1.4 *	21.7
	IGF-1-user	4.3 ± 1.2	5.1 ± 1.5 *	18.6
**GPx**	Non-user	6.7 ± 0.7	8.5 ± 1.0 *	26.8
**(mU/mL)**	GH-user	7.3 ± 0.8	9.4 ± 0.8 *‡	28.7
	IGF-1-user	6.9 ± 0.8	8.4 ± 0.9 *	21.7
**CAT**	Non-user	8.2 ± 0.7	8.7 ± 0.8	6.1
**(mU/mL)**	GH-user	8.3 ± 0.6	8.5 ± 0.8	2.4
	IGF-1-user	8.2 ± 0.7	8.7 ± 0.7	6.1
**GLU**	Non-user	93.4 ± 4.3	106.3 ± 4.9 *	13.8
**(mg/dL)**	GH-user	94.3 ± 3.5	115.1 ± 5.7 *‡	22.1
	IGF-1-user	93.5 ± 4.1	102.4 ± 5.1 *	9.5

8-OHdG: 8-hydroxy-2-deoxyguanosine, MDA: malondialdehyde, NO: nitric oxide, GPx: glutathione peroxidase, CAT: catalase, GLU: glutamate. * denotes significant differences as compared with pre-test, † denotes significant differences as compared with GH-user, ‡ denotes significant differences as compared with non-user and IGF-1 user. Values are mean ± SD.
